# Hospital-based influenza morbidity and mortality surveillance system for influenza-like illnesses: a comparison with national influenza surveillance systems

**DOI:** 10.1111/irv.12175

**Published:** 2013-09-11

**Authors:** Yu Bin Seo, Joon Young Song, Hee Jin Cheong, Young Duck Cho, Seong-Heon Wie, Hye Won Jeong, Woo Joo Kim

**Affiliations:** a Division of Infectious Diseases, Department of Internal Medicine, Korea University College of MedicineSeoul, Korea; b Asian Pacific Influenza Institute (APII)Seoul, Korea; c Department of Emergency Medicine, Korea University College of MedicineSeoul, Korea; d Catholic University Medical College, St. Vincent HospitalSuwon, Korea; e Division of Infectious Diseases, Department of Internal Medicine, Chungbuk National University College of MedicineCheongju, Korea

**Keywords:** Influenza, influenza-like illness, Korea, surveillance

## Abstract

The Hospital-based Influenza Morbidity and Mortality (HIMM) surveillance system is an emergency room (ER)-based influenza surveillance system in Korea that was established in 2011. The system was established under the assumption that integrated clinical and virologic surveillance could be performed rapidly and easily at seven tertiary hospitals' ER. Here, we assessed the correlation between data generated from the HIMM surveillance system and the Korean national influenza surveillance systems during the 2011–2012 influenza season using cross-correlation analysis and found strong correlations. Rapid antigen-test-based HIMM surveillance would predict the start of influenza epidemic earlier than pre-existing influenza-like-illness-based surveillance.

## Introduction

Public influenza surveillance systems are important for early detection and rapid response to an epidemic. In Korea, the two separate national surveillance networks that report on the circulation of influenza are the outpatient influenza-like illness (ILI) surveillance network (Korean Influenza Surveillance System, KISS) and the laboratory virus surveillance system (Korean Influenza and Respiratory Viruses Surveillance Scheme, KINRESS). In 2011, the Trans-government Enterprise of Pandemic Influenza in Korea (TEPIK) developed another hospital-based influenza surveillance system, named “Hospital-based Influenza Morbidity and Mortality” (HIMM). The HIMM consists of an integrated clinical and laboratory surveillance system, in which seven nationwide tertiary teaching hospitals collect data of patients who visit the emergency room with ILI. This system aims to monitor not only influenza activity but also influenza severity, such as hospitalization, complication and mortality, which cannot be reflected by national surveillance systems. The objective of this study was to compare the surveillance data of HIMM with KISS and KINRESS in its first year of operation during the 2011–2012 influenza season.

## Methods

Analyses were performed according to the week of the year with week 1 defined as starting with the week (from Monday through Sunday) in which the January 1 is allocated. The study period was from October 2, 2011, through June 30, 2012. We restricted our analysis to the period during which HIMM surveillance was initiated, from week 41 of 2011 through week 26 of 2012.

In the HIMM surveillance network, participating sites prospectively entered the data of patients who visited the emergency room with an ILI (defined as sudden onset of fever ≥37·8°C accompanied by ≥1 respiratory symptoms such as cough, sore throat, or rhinorrhea) into the Internet database. The weekly incidence of ILI per 1000 patients who visited the ER was calculated from the database. A rapid antigen test (SD Bioline rapid influenza test, Standard Diagnostics, Inc., Yongin City, Korea ) for influenza virus was performed using the respiratory samples that were collected by nasopharyngeal swab or aspirate, and throat swab if the patient consented to the test. During the study period, samples were taken from 41·3% of 10 872 patients presenting with ILI. The influenza test positivity was defined as the ratio of the number of positive cases divided by the total number of tests. RAT positivity was reported weekly.

To compare the HIMM data, the data from the two national influenza surveillance systems were obtained from the Korean National Institute of Health (KNIH). Both HIMM and KINH use same definition of ILI. KISS surveillance consists of a network of about 800 public health centers and private clinics that record the weekly proportion of patients who have ILI symptoms.^1^ KISS reports the weekly incidence of ILI per 1000 outpatients. KINRESS surveillance consists of about 100 hospitals that obtain the respiratory specimens by nasopharyngeal swab, nasopharyngeal aspirate, or throat swab from patients presenting with ILI. Samples are forwarded to KNIH, where viruses are characterized by self-developed multiplex reverse-transcriptase polymerase chain reaction (RT-PCR). The KINRESS reports weekly positivity of influenza tests along with other respiratory viruses, including respiratory syncytial virus, parainfluenza virus, adenovirus, human rhinovirus, human metapneumovirus, human coronavirus and human bocavirus.

The influenza epidemic period was defined as the week in which the ILI rate crossed a certain value based on a seasonal threshold set by the KNIH. Baseline ILI rate was calculated as the average of the values of the ILI rate during the pre-epidemic period. Scatter plots were constructed to compare HIMM ILI surveillance data with KISS surveillance data. From these comparisons, Pearson's correlation coefficients (*R*) were calculated, and a relationship is reported as significant at *P* < 0·05. Correlation coefficients were subsequently calculated for regional HIMM and KISS surveillance data to monitor regional influenza trends. We undertook an additional correlation analysis to determine whether HIMM RAT surveillance was correlated with KINRESS RT-PCR surveillance. Data analysis was performed using spss, version 10·0 (SPSS Inc., Chicago, IL, USA). The study was approved by the ethics committee of each institution involved and was conducted in accordance with the Declaration of Helsinki and Good Clinical Practice.

## Results

**Figure 1:**
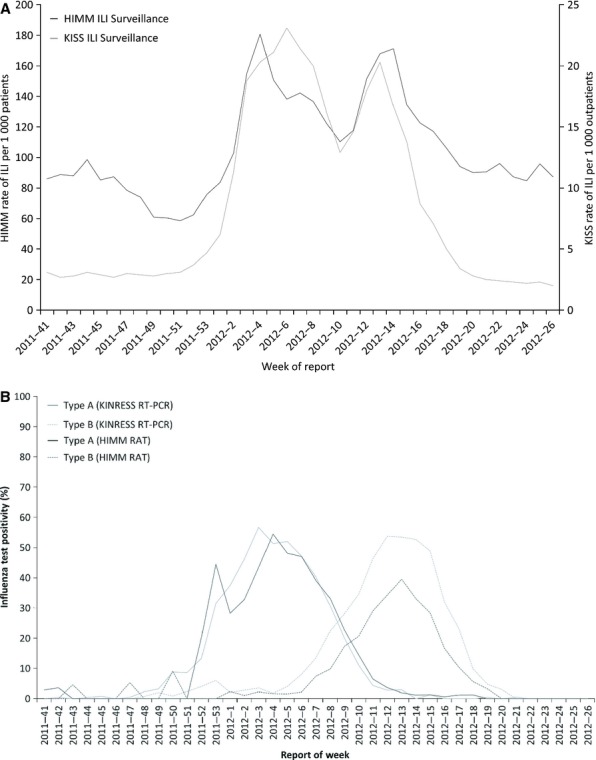
Comparison The Hospital-based Influenza Morbidity and Mortality (HIMM) surveillance system with national influenza surveillance system from October 2, 2011, through June 30, 2012.

During the study period, HIMM ILI surveillance showed a strong temporal correlation with KISS surveillance. Simple inspection revealed broad similarity in terms of timing of seasonal peaks (Figure 1). The correlation between HIMM ILI surveillance and KISS surveillance was 0·886. In addition, the initial upswing above the ILI baseline occurred at the same time from week 53 in both KISS and HIMM ILI surveillance systems. Although the weekly ILI rate patterns of the two surveillance systems closely resembled each other, the HIMM surveillance showed a higher baseline rate of ILI. The mean baseline rate of ILI before the influenza epidemic was 77·4 of 1000 emergency patients in the hospital-based influenza morbidity and mortality and 2·9 of 1000 outpatients in the Korean influenza surveillance.

When compared by region, a high correlation was found in the regions in which HIMM hospitals were located. The correlation between regional HIMM ILI and KISS surveillance ranged from 0·717 in Gyeonggi to 0·894 in Seoul (Table 1). The mean correlation for these comparisons was 0·814. Each site showed a relatively stable contribution to the total ILI cases each week.

**Table 1:** Pearson's correlation coefficient matrix of data from regional KISS and HIMM ILI surveillance

KISS surveillance	HIMM site	Correlation coefficient
Seoul	Korea University Guro Hospital	0·894
	Hallym University	0·850
Incheon	Inha University	0·853
Gyeonggi	Korea University Ansan Hospital	0·717
	St. Vincent's Hospital	0·830
Gangwon	Yonsei University Wonju College	0·772
Chungbuk	Chungbuk National University	0·780

The weekly influenza cases detected by HIMM RAT and KINRESS RT-PCR surveillance during the study period are shown in Figure 1. Two epidemic peaks were noted. Influenza A was predominant during the first epidemic peak, while influenza B was predominant during the second peak. There was a strong correlation between the two surveillance systems (*R* = 0·961). The initial rise occurred earlier in KINRESS surveillance from week 51 and in HIMM from week 52. In addition, HIMM RAT surveillance and KINRESS RT-PCR surveillance, respectively, showed strong correlation with HIMM ILI surveillance and KISS ILI surveillance (HIMM ILI versus HIMM RAT, *R* = 0·742; KISS ILI versus KINRESS RT-PCR, *R* = 0·935).

## Discussion

HIMM ILI and HIMM RAT surveillance systems were established under the assumption that effective influenza surveillance could be performed easily and rapidly at the tertiary hospital emergency room level. This study provided a report of the first year of HIMM surveillance and assessed the correlation with the two Korean national influenza surveillance systems.

The comparison between HIMM ILI and national ILI surveillance demonstrated the reliability of tertiary hospitals' ER-based surveillance to monitor influenza activity. KNIH collects data from sentinel practitioners and disseminates the results weekly by an online report, and this process takes about one week. In the first operating year, HIMM collected data and released the reports at the same time as KNIH. However, HIMM could achieve daily analysis of ILI rates by asking sentinel sites, and this suggests that HIMM could identify the daily influenza activity. This may provide an advantage of HIMM ILI surveillance compared with other established small-scale influenza networks.^2^,^3^

It is interesting that HIMM surveillance showed a higher baseline ILI rate than national ILI surveillance. Because the cost of ER care in Korea is not as expensive as in other countries and over-the-counter drugs are not available in supermarkets, many patients tend to visit the ER when local clinics are closed, regardless of the severity of the disease. Therefore, patients with ILI are always found visiting the ER.

The ILI rates need to be interpreted with caution because they may not represent true cases of influenza. Additional laboratory surveillance is important to initiate proper preventive measures with confidence. For this reason, the results produced by virologic surveillance should be available with as little delay as possible. Although virologic surveillance can be carried out by various techniques, RT-PCR is commonly used in most influenza surveillance systems. However, it is labor-intensive and time-consuming. The strong correlation between RAT-confirmed positivity by HIMM and RT-PCRs by KINRSS supports the use of RATs for evaluating real-time confirmed influenza activity. Previous studies revealed the simplicity and reliability of RAT for surveillance of influenza epidemics, and our study supports these results.^4^,^5^

Early detection of an epidemic is important to prepare for the potentially serious threat to public health. Although few studies have evaluated the timeliness of ILI surveillance compared with laboratory surveillance, ILI surveillance has been reported to detect the onset of seasonal influenza before an increase in laboratory notification.^6^,^7^ However, surveillance systems for early detection may be affected differently by geography and methodology. In our analysis, an upswing of HIMM RAT-based surveillance occurred at least one week earlier than in the national ILI surveillance. This finding suggests that HIMM RAT surveillance could be useful to detect the early signs of an influenza epidemic.

We note that our study had several limitations. Compared with KISS surveillance, the baseline ILI rates of HIMM surveillance showed fluctuation during the initial operating period, and this may have affected the results. This fluctuation may be due to the immaturity of data collection at that time. Another limitation was the limited information of epidemiological features in national surveillance systems, so comparison along with age, sex, influenza vaccination, and comorbidities was not available.

In conclusion, the HIMM surveillance system has some strengths compared with national influenza surveillance. HIMM surveillance can monitor influenza circulation based on a small number of nationwide tertiary institutions, so more rapid data processing and reporting are possible. Moreover, it demonstrated the possibility of detecting early signals of influenza epidemics by using simple and quick RATs. HIMM surveillance can also monitor severity of influenza infection by collecting the data of the hospitalization, intensive care unit admission, complications, and mortality, which cannot be reflected by national surveillance system. We hope that HIMM surveillance will prove to be an excellent complementary tool to national influenza surveillance systems in the future.
